# Active Droplet Formation in a Microfluidic Cross-Junction Using Stacked Piezoelectric Actuators

**DOI:** 10.3390/mi17091069

**Published:** 2026-09-09

**Authors:** He Yang, Baokai Huang, Wen Wang, Zhanfeng Chen, Keqing Lu

**Affiliations:** 1 School of Aerospace Engineering and Applied Mechanics, Tongji University, Shanghai 200092, China; 2 Shanghai Key Lab of Vehicle Aerodynamics and Vehicle Thermal Management Systems, Shanghai 200092, China; 3 School of Mechanical Engineering, Hangzhou Dianzi University, Hangzhou 310018, China

**Keywords:** droplet formation, active control, on-demand droplet generation, piezoelectric actuators, microfluidics

## Abstract

On-demand droplet formation is of crucial importance to the engineering applications of droplet microfluidics. This work presents an experimental investigation on active control of droplet formation using stacked piezoelectric actuators. Two stacked piezoelectric actuators are placed adjacent to the continuous phase channel, producing periodic excitations on the continuous phase flow. It is found that droplet formation greatly depends on the excitation frequency and voltage. Droplet formation synchronizes piezoelectric excitation at a small excitation frequency, i.e., droplet formation frequency equals excitation frequency and its subharmonics. Beyond a critical value of the excitation frequency, a neglected effect of excitation frequency on droplet generation is observed. The droplet generation frequency could be increased up to ~2.6 times that without excitation. The droplet generation frequency exhibits a stepwise increase with rising excitation voltage. When the droplet formation frequency equals the excitation frequency, droplet formation undergoes filling, necking, and pinching-off. When the droplet formation frequency is half of the excitation frequency, additional refilling and re-necking stages are observed. The regime diagram of the droplet formation frequency in the synchronization mode is presented. The scaling of the generated droplet length is deduced. Since periodic excitations are employed on the continuous phase flow, the proposed active control method could minimize the detrimental impact on biochemical reagents within droplets.

## 1. Introduction

Droplet microfluidics has emerged as a fascinating tool across a wide range of fields, such as single-cell analysis [1,2,3], protein aggregation [4,5,6], and drug delivery [7,8,9,10]. It enables massive production of microscale droplets from immiscible fluids, which is beneficial to the manipulation of small-volume reagents in isolated compartments [10,11,12,13]. One of the key requirements for droplet microfluidics is on-demand production of monodispersed droplets, which is crucial to the quantitative analysis of biochemical reactions and programmable synthesis of nanoparticles [8,13].

Many microfluidic devices have been developed to generate uniform droplets, both passively and actively. Passive devices produce droplets through innovative design of channel geometries, including T-junctions [14,15,16,17,18,19,20,21], co-flowing channels [22], and cross-junctions [23,24,25,26,27,28,29,30]. Once passive devices are fabricated, the droplet size can be altered by adjusting the flow rates of the dispersed phase and continuous phase fluids. However, the response time can be quite long, e.g., several or even tens of minutes [8,29,31]. Due to the simplicity of passive methods, the flow physics behind droplet formation has been extensively investigated. Several modes of droplet formation have been revealed, including squeezing, dripping and jetting [23,27,28,32,33,34,35,36,37,38]. Channel confinement, also referred to as the squeezing mechanism, is responsible for droplet generation in squeezing mode [35,39,40], while Rayleigh–Plateau instability is the dominant mechanism for other modes [8,32,41].

In comparison with passive approaches, active methods have faster response and higher uniformity. Droplet formation in active devices can be adjusted by employing external actuators, which can be classified as electrical, magnetic, thermal, mechanical, and acoustic [8,31,42]. Electrical devices apply high-voltage (~10 kV) electric fields on the dispersed phase, introducing an additional electric force on the liquid interface [43,44,45,46]. Permanent magnets or electromagnets could exert magnetic fields on ferromagnetic fluids, generating ferromagnetic droplets [47,48]. Thermal methods employ a focused laser to locally heat the fluids, which could change fluid viscosity and interfacial tension [31]. However, droplet formation in squeezing mode is less sensitive to temperature, since pressure forces are much larger than viscous forces at small capillary number [31]. Mechanical approaches use membrane valves or a mechanical vibrator to physically deform the channel or introduce pressure fluctuations to the dispersed phase [31,49]. Acoustic control integrates interdigitated transducers on the microfluidic chip, producing surface acoustic waves (SAWs) [50,51]. The SAW imposes acoustic forces on the thinning neck during droplet formation. Therefore, active control introduces additional excitation on the interface between the dispersed and continuous phases, manipulating the production of monodispersed droplets. However, most of the active actuators are exerted on the dispersed phase, which may impair the bioactivity of the reagents.

Previous numerical investigations [52,53] have shown the feasibility of active droplet generation with pulsatile flow of the continuous phase. In this work, we present an experimental achievement for continuous phase perturbations. Two stacked piezoelectric actuators are used to exert excitations on the continuous phase. The proposed active method could achieve on-demand control of droplet formation, minimizing the detrimental impact on biochemical reagents within droplets. The dependence of droplet formation on excitation frequency and voltage is explored at various flow rates of the dispersed phase. The droplet generation frequency and size in synchronized regimes are analyzed. Then, the droplet formation process and physical mechanism in different synchronized regimes is discussed. Finally, we present the regime diagram of the droplet generation frequency and the scaling equation of the generated droplet length.

## 2. Experimental Methods

### 2.1. Design of the Microfluidic Device

**Figure 1:**
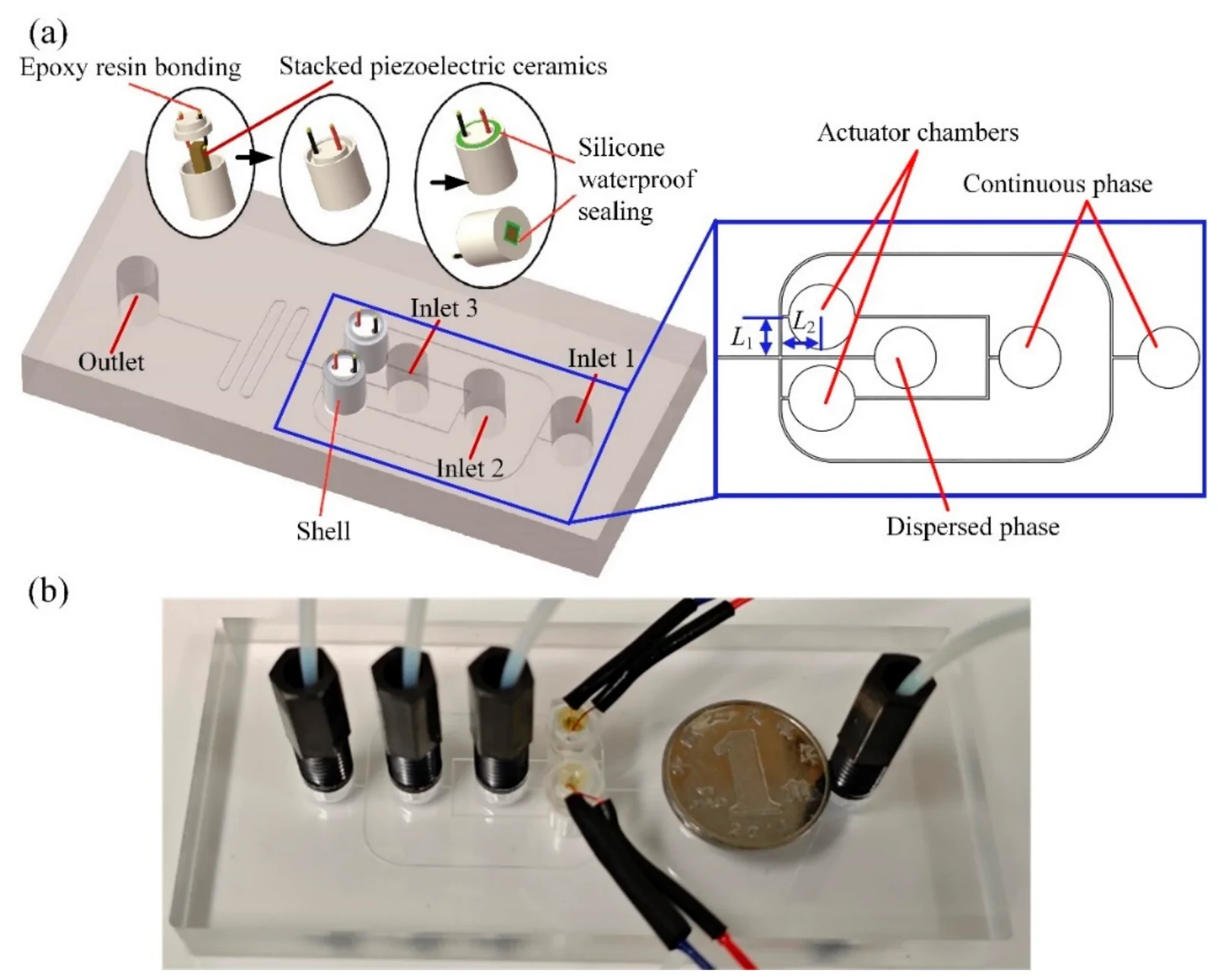
(**a**) Schematic assembly and (**b**) photograph of the microfluidic chip with stacked piezoelectric actuators.

A schematic diagram of the microfluidic chip with piezoelectric actuators is shown in Figure 1. It consists of three inlets, a cross junction, two excitation chambers and an outlet. The microfluidic channel has a depth and width of *h* = *w* = 200 μm. The continuous phase is injected through inlet 1, while the dispersed phase flows through inlet 3. The droplets are generated at the cross junction of the microfluidic chip. Two piezoelectric actuators with shells are placed into two chambers, which are connected to the continuous phase channels. Each excitation chamber has a diameter of 6 mm, and the chamber center has a lateral displacement of *L*_1_ = *L*_2_ = 5 mm away from the cross junction. Before experiments, the continuous phase fluid is also injected into the excitation chambers through inlet 2 to fully drain the air. The polymethyl methacrylate (PMMA) chip is fabricated by a high-precision CNC machine (DA-600T, OSDIOU, Dongguan, China).

The assembly of the piezoelectric actuators on the microfluidic chip is illustrated in Figure 1a. Two stacked piezoelectric actuators (PSt100/1.65 × 1.65/5H, Coremorrow, Harbin, China) are used for active control of droplet formation. Each actuator has a size of 1.65 mm × 1.65 mm × 5 mm and a maximum input voltage of 100 V. The piezoelectric stack actuators are placed into two shells. Each shell has an upper cover and a circumferential cover. The piezoelectric stack actuators are adhered to the upper cover and then assembled into the circumferential cover. The waterproof silicone adhesive is used to seal the gap between the piezoelectric stack actuator and the shell, to avoid liquid leakage. Finally, each shell is mounted on the excitation chamber. As the piezoelectric stack actuators deform, the fluid in the excitation chamber is pushed outwards or pulled inwards, producing pulsative flow in the continuous phase microchannels.

### 2.2. Experimental Setup and Fluid Properties

**Figure 2:**
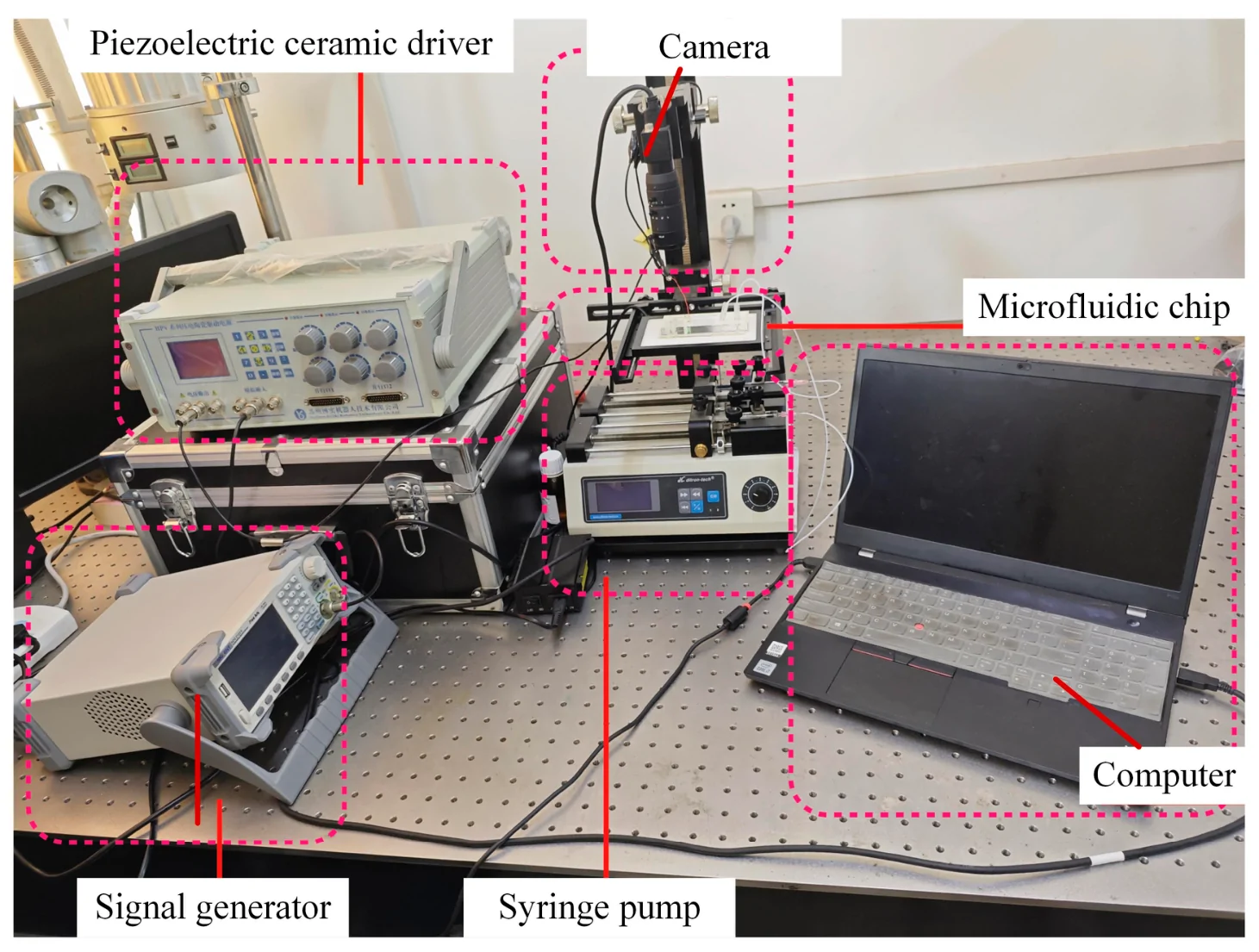
Photograph of the experimental setup.

The experimental setup consists of two syringe pumps, a microfluidic chip, a signal generator, a piezoelectric ceramic driver, a collector and an industrial camera, as shown in Figure 2. Syringe pumps (LSP02-3B, Longer, Baoding, China) are used for the injection of the dispersed and continuous fluids into the microfluidic chip. The signal generator (SDG2122X, Siglent, Shenzhen, China) is employed to produce the sinusoidal signal. The piezoelectric ceramic driver (HPV-3C0150A0500, Boshi, Suzhou, China) is used to amplify the signal, driving the piezoelectric stack actuators synchronously and in phase. Under the excitation of the piezoelectric actuators, active control of droplet formation at the cross junction is achieved. The photographs of droplet formation under each excitation were captured for 30 s using an industrial camera (GP-660V, Gaopin, Suzhou, China). The droplet generation frequency is determined by synchronization of the adjusted camera frame rate and droplet movement. The droplet length is measured by the software ImageJ (version: 1.52a) and averaged every five sequential droplets.

To achieve periodic excitations in this work, a sinusoidal signal is used to drive the piezoelectric stack actuators, which can be expressed by

*E*(*t*) = *E*_0_sin(2π*f*_e_*t*) + *E*_0_ (1)

where *E*_0_ and *f*_e_ denote the excitation voltage and frequency, respectively. *t* represents the time.

The axial displacement *ε*(*t*) of the piezoelectric stack actuator can be described by

*ε*(*t*) = *ε*_0_sin (2π*f*_e_*t*) + *ε*_0_ (2)

where *ε*_0_ = *d*_33_*E*_0_, and *d*_33_ is the effective displacement coefficient, *d*_33_ = 4.19 × 10^−8^ m/V, provided by the supplier (Coremorrow, Harbin, China).

The motion of the piezoelectric actuator leads to a change in the fluid volume in the chamber, which can be expressed by

*V*_e_(*t*) = *ε*(*t*)*A* = *d*_33_*E*_0_sin(2π*f*_e_*t*)*A* + *d*_33_*E*_0_*A* (3)

where *A* is the cross-sectional area of the piezoelectric stack actuator.

The flow rate in the chamber caused by the piezoelectric excitation can be calculated by

(4) $$Q_{e}(t)=\frac{dV_{e}(t)}{dt}=2\pi f_{e}Ad_{33}E_{0}\cos(2\pi f_{e}t)$$

Thus, the flow rate in each continuous phase microchannel is given by

(5) $$\frac{Q_{\mathrm{ce}}(t)}{2}=\frac{Q_{c}}{2}+Q_{e}(t)=\frac{Q_{c}}{2}+2\pi f_{e}Ad_{33}E_{0}\cos(2\pi f_{e}t)$$

where *Q*_c_ is the flow rate at the inlet of the continuous phase. This fluctuation in the flow rate of the continuous phase could change local force balance during droplet formation. As a result, the droplet formation can be controlled by the excitation of the piezoelectric actuators.

The fluid properties of the dispersed and continuous phases are presented in Table 1. The dispersed phase fluid used in this work is liquid paraffin (Aladdin, Shanghai, China) with a density of 0.84 g/cm^3^ and a dynamic viscosity of 16.07 mPa·s, and the continuous phase is distilled water with a density of 1.0 g/cm^3^ and a dynamic viscosity of 0.89 mPa·s. The interfacial tension between the dispersed phase and the continuous phase is 46.7 × 10^−3^ N/m. For droplet visualization, the dispersed phase was dyed with 0.02%w/w Solvent Blue (Macklin, Shanghai, China).

## 3. Results and Discussion

### 3.1. Dependence of Droplet Formation on Excitation Frequency

**Figure 3:**
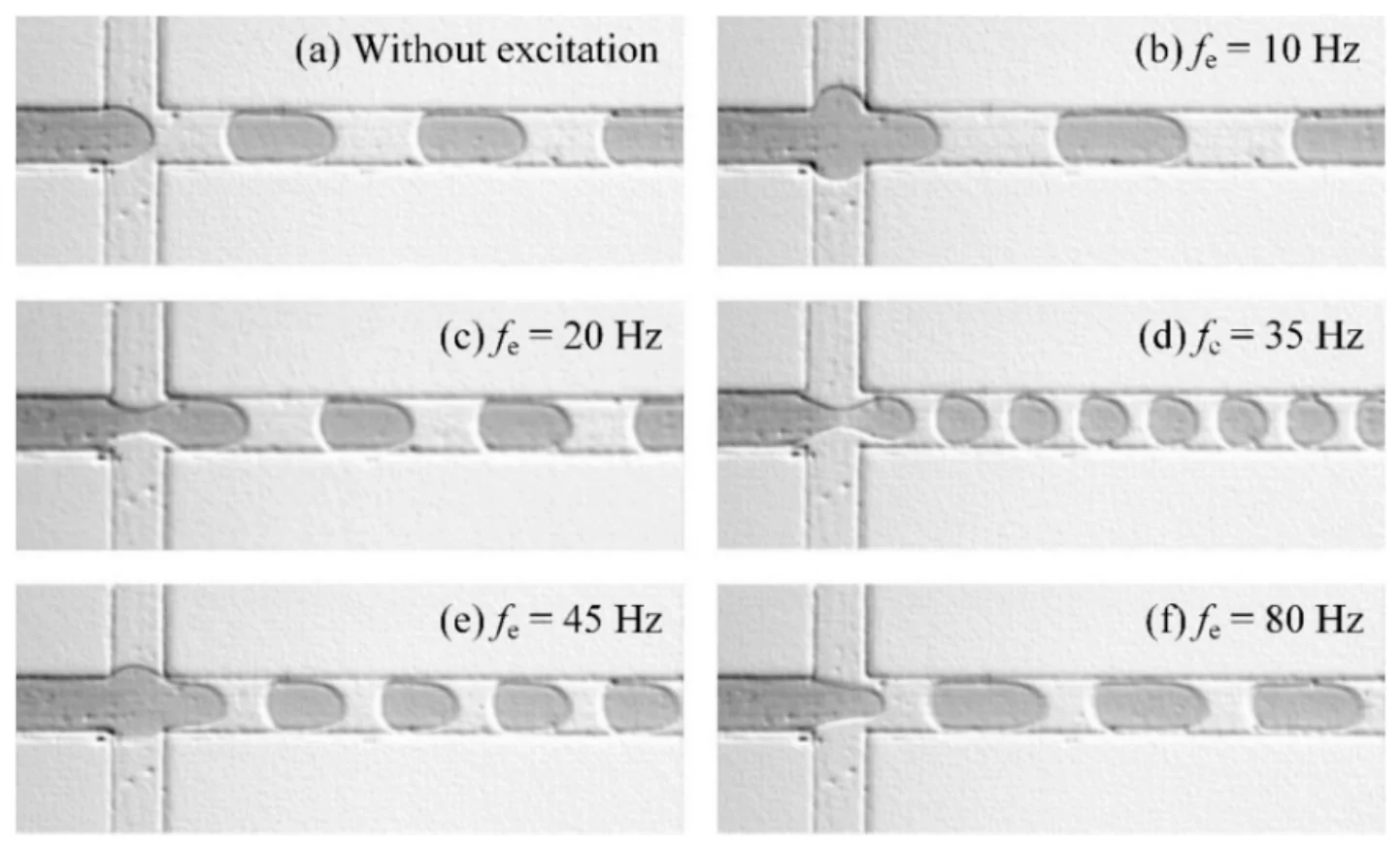
Photographs of droplet formation at various excitation frequencies (*Q*_d_ = *Q*_c_ = 700 µL/h, *E*_0_ = 40 V).

Under the excitation of the piezoelectric actuators, droplet generation can be actively manipulated. Figure 3 presents photographs of droplet formation at various excitation frequencies. These photographs were chosen based on the variation tendency of droplet formation. The flow rates of the dispersed phase and the continuous phase are fixed at *Q*_d_ = *Q*_c_ = 700 µL/h. The piezoelectric stack actuators are driven with an excitation voltage of *E*_0_ = 40 V. In the absence of piezoelectric excitation, droplets are formed with a length of *l*_d_/*w* = 2.25 at a frequency of *f*_d_ = 14 Hz. Once the piezoelectric actuators are driven with an excitation frequency of *f*_e_ = 10 Hz, droplets are produced at a frequency of *f*_d_ = 10 Hz, and the droplet length is 20.79% longer than that without excitation. When the excitation frequency increases to *f*_e_ = 20 Hz, smaller droplets with a length of *l*_d_/*w* = 1.77 are formed at a frequency of *f*_d_ = 20 Hz. At *f*_e_ = 35 Hz, the droplet length is reduced to *l*_d_/*w* = 1.07, which is roughly half that without excitation. The droplet generation frequency is still equal to the excitation frequency of the piezoelectric actuators, i.e., *f*_e_ = *f*_d_ = 35 Hz. However, as the excitation frequency further rises to *f*_e_ = 45 Hz, droplets are formed at a frequency of *f*_d_ ≈ (1/2) *f*_e_. This indicates that one droplet is produced every two periods of the excitation. The droplet length increases to *l*_d_/*w* = 1.63, due to the reduced generation frequency. Further, at *f*_e_ = 80 Hz, droplets are generated with a length of *l*_d_/*w* = 2.38 at a frequency of *f*_d_ = 13 Hz, which is roughly equal to that without excitation. Note that droplet formation is mainly in squeezing mode. This is different from the numerical investigations of active droplet formation with pulsatile flow [52], in which jetting and thread flow are also observed. The difference can be ascribed to the low flow rates of the dispersed and continuous phases used in this work.

**Figure 4:**
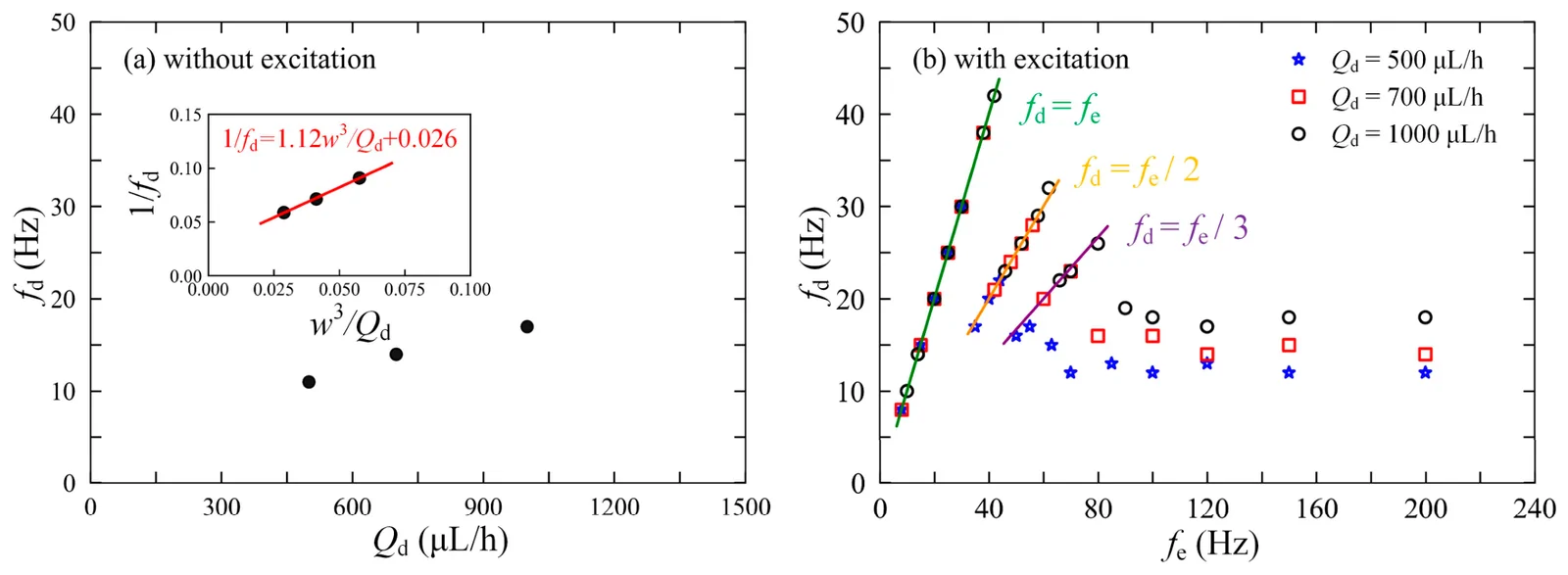
Effect of the excitation frequency on the droplet generation frequency (*Q*_c_ = 700 µL/h, *E*_0_ = 40 V): (**a**) without excitation, (**b**) with excitation.

Figure 4 presents the dependence of the droplet generation frequency on the excitation frequency of the piezoelectric actuators at various flow rates of the dispersed phase. Three flow rates of the dispersed phase are examined, including *Q*_d_ = 500 µL/h, *Q*_d_ = 700 µL/h, and *Q*_d_ = 1000 µL/h. The flow rate of the continuous phase is fixed at *Q*_c_ = 700 µL/h, and the excitation voltage is chosen as *E*_0_ = 40 V. Without excitation, the droplet generation frequency varies from *f*_d_ = 11 Hz to *f*_d_ = 17 Hz with the increase in the flow rate of the dispersed phase from *Q*_d_ = 500 µL/h to *Q*_d_ = 1000 µL/h (Figure 4a). Note that 1/*f*_d_ exhibits a linear relationship with *w*^3^/*Q*_d_, i.e., 1/*f*_d_ = 1.12*w*^3^/*Q*_d_ + 0.026. The dimensionless droplet volume in a cross-junction has a linear scaling with the flow rate ratio of the dispersed phase and the continuous phase [25,33], which is given by

(6) $$\frac{V_{d}}{w^{3}}=\alpha +\beta \frac{Q_{d}}{Q_{c}}$$

where *α* and *β* are coefficients, which depend on the channel geometry and fluid properties.

Based on mass conservation, the droplet volume *V*_d_ has a relationship with the flow rate of the dispersed phase *Q*_d_ and the droplet generation frequency *f_d_*, which can be expressed by

(7) $$V_{d}=\frac{Q_{d}}{f_{d}}$$

By combining Equations (6) and (7), we have

(8) $$\frac{1}{f_{d}}=\alpha \frac{w^{3}}{Q_{d}}+\beta \frac{w^{3}}{Q_{c}}$$

Once excited, the droplet generation frequency *f*_d_ presents a linearly rising tendency with the increase in the excitation frequency *f*_e_ before reaching a steady state. Specifically, at small *f*_e_ (8 ~ 80 Hz), the droplet generation frequency equals the excitation frequency and its subharmonics, i.e., *f*_d_ = *f*_e_, *f*_d_ = *f*_e_/2, and *f*_d_ = *f*_e_/3. This indicates that the droplet generation frequency is synchronized with the excitation frequency. In contrast, the droplet generation at large *f*_e_ (>80 Hz) is independent of the excitation frequency. This is similar to the findings in the investigation of droplet generation in co-flow with a mechanical vibrator on the dispersed phase [49]. Beyond a critical frequency, the vibration frequency exhibits a weak effect on droplet generation [49]. In synchronization mode, the droplet generation frequency equals the vibration frequency [49]. However, subharmonic frequency synchronization is not observed in their work. Note that the maximal droplet generation frequency is *f*_d_ = 30 Hz at *Q*_d_ = 500 µL/h, *f*_d_ = 38 Hz at *Q*_d_ = 700 µL/h, and *f*_d_ = 42 Hz at *Q*_d_ = 1000 µL/h. This indicates that the droplet generation frequency could be increased to ~2.6 times that without excitation. Moreover, all *f*_d_ for three *Q*_d_ fit well in synchronization mode. This suggests that the droplet generation frequency could be independent of the flow rate if the excitation frequency remains unchanged. Therefore, the droplet generation frequency could be precisely controlled with excitations of the stacked piezoelectric actuators.

**Figure 5:**
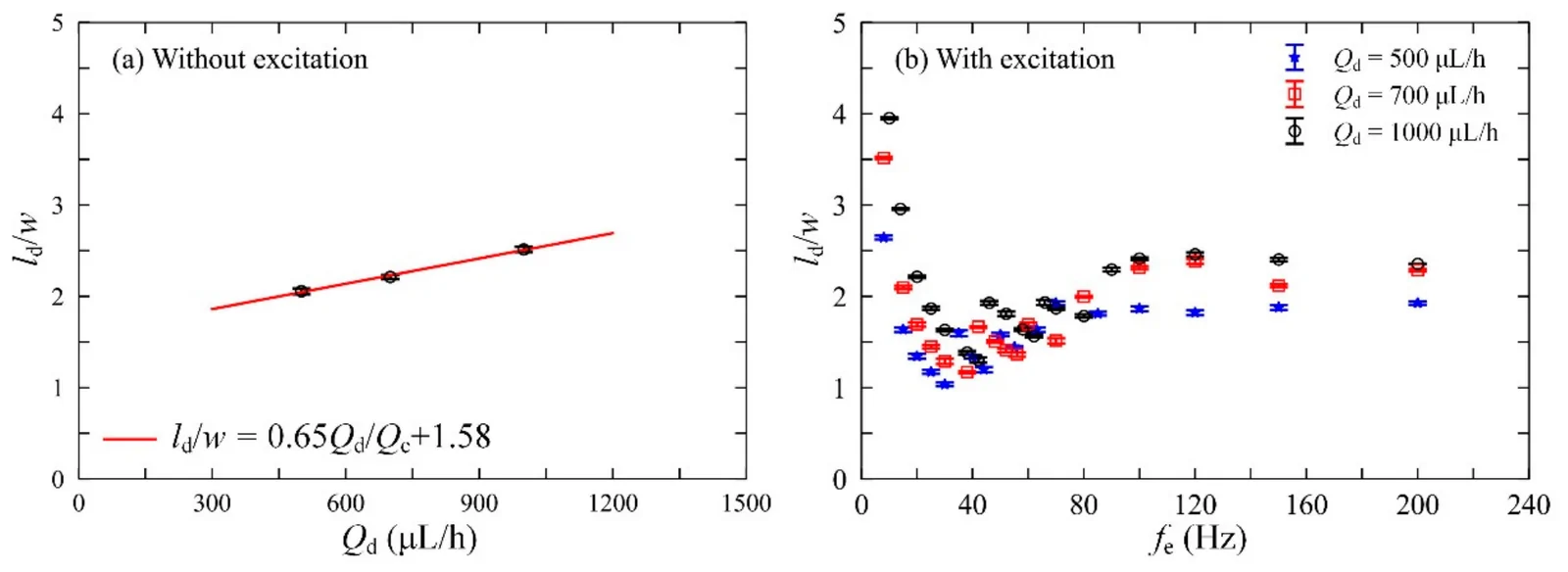
Effect of excitation frequency on droplet length. (*Q*_c_ = 700 µL/h, *E*_0_ = 40 V): (**a**) without excitation, (**b**) with excitation.

Figure 5 presents the averaged value and standard deviation of the dimensionless droplet length at various excitation frequencies. The flow rate of the continuous phase is fixed at *Q*_c_ = 700 µL/h and the piezoelectric stack actuators are driven with an excitation voltage of *E*_0_ = 40 V. Without excitation, the droplet length exhibits a linear relationship with the flow rate of the dispersed phase, i.e., *l*_d/_*w* = 0.65*Q*_d_/*Q*_c_ + 1.58 (Figure 5a). This linear scaling is consistent with numerical investigations of droplet generation in cross-junctions with the lattice Boltzmann method [40]; the relationship between the dimensionless droplet length and the flow rate ratio is given by

(9) $$\frac{l_{d}}{w}=\varepsilon +\varphi \frac{Q_{d}}{Q_{c}}$$

where *ε* and *φ* are the coefficients. The difference of the fitting coefficients between this work and numerical investigations can be ascribed to the difference in channel geometry and fluid properties.

With excitation, the droplet length for all *Q*_d_ gradually decreases to a minimal value at small excitation frequency and rises to a steady value for *f*_e_ > 100 Hz. This indicates that the excitation frequency has a significant effect on the droplet size at small excitation frequency. The droplet length at *Q*_d_ = 1000 µL/h reduces from *l*_d_/*w* = 3.95 at *f*_e_ = 10 Hz to *l*_d_/*w* = 1.30 at *f*_e_ = 42 Hz. The ascending tendency of the droplet length can be ascribed to the increase in the droplet generation frequency (Figure 4b). The maximal and minimal droplet lengths with excitation are 156.75% and 51.59% that without excitation, respectively. This indicates that the droplet length could be adjusted over a large range at a fixed flow rate.

### 3.2. Dependence of Droplet Formation on Excitation Voltage

**Figure 6:**
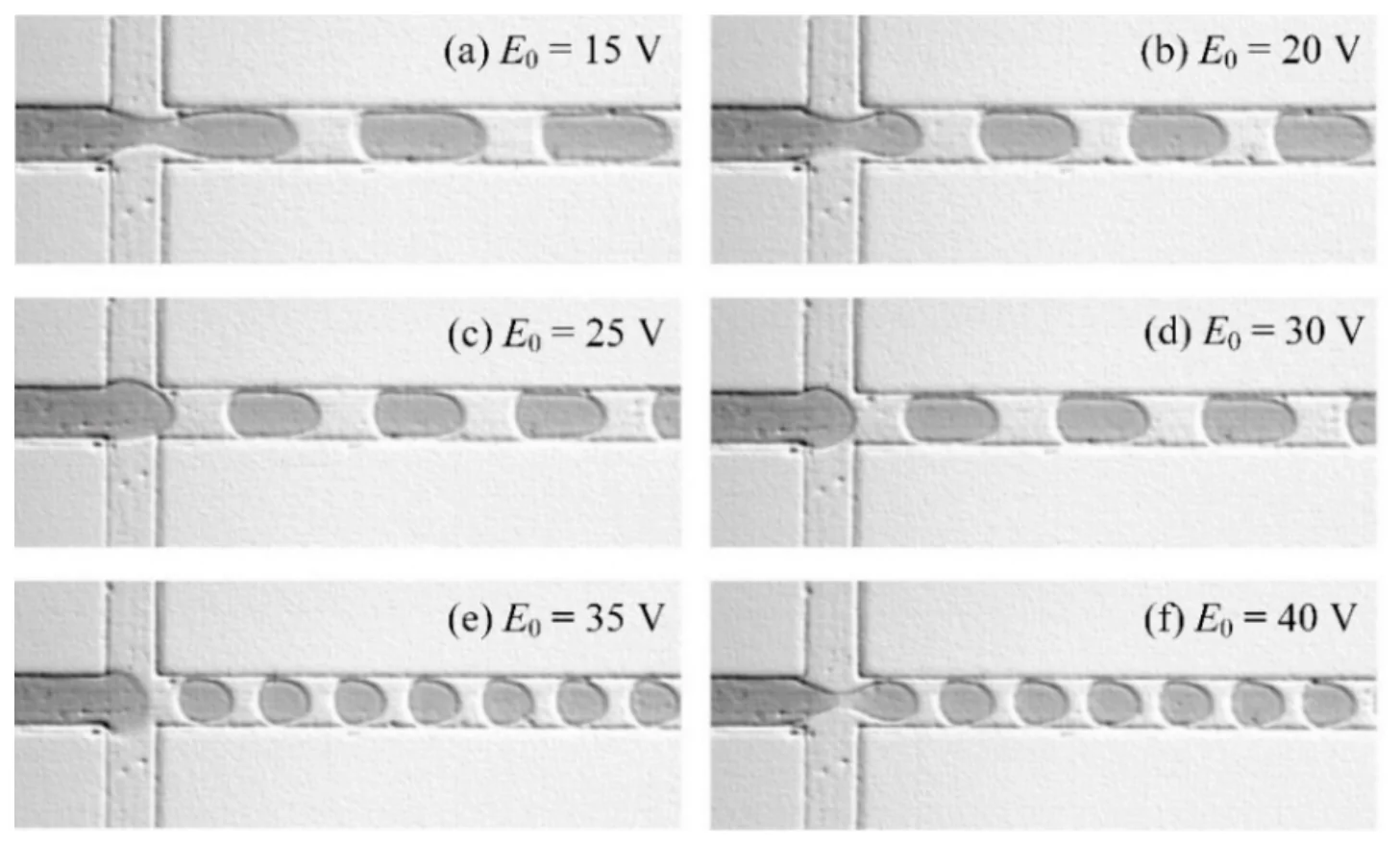
Photographs of droplet formation at different excitation voltages (*Q*_d_ = *Q*_c_ = 700 µL/h, *f*_e_ = 36 Hz).

The excitation voltage *E*_0_ determines the displacement of the piezoelectric stack actuators, which affects the fluctuation of the flow rate of the continuous phase. Figure 6 presents photographs of droplet formation at various excitation voltages. The flow rates of the continuous phase and the dispersed phase at the inlets are fixed at *Q*_c_ = *Q*_d_ = 700 μL/h. The excitation frequency is chosen as *f*_e_ = 36 Hz, while the excitation voltage is varied from *E*_0_ = 15 V to *E*_0_ = 40 V. It can be observed that the droplet length at an excitation voltage of *E*_0_ = 15 V is *l*_d_/*w* = 2.89, which is the largest among the range of the examined voltages. As the excitation voltage rises to *E*_0_ = 20 V, the droplet length reduces to *l*_d_/*w* = 2.06. The droplet length remains roughly unchanged with the increase in the excitation voltage from *E*_0_ = 20 V to *E*_0_ = 30 V. Further, at *E*_0_ = 35 V and *E*_0_ = 40 V, the droplet length becomes *l*_d_/*w* = 1.23. Also, it is evident that the droplet generation frequency at *E*_0_ = 35 V and *E*_0_ = 40 V is highest among the excitation voltage range of *E*_0_ = 15 V ~ 40 V. Therefore, it can be inferred that the droplet length may exhibit a step reduction with the increase in the excitation voltage.

**Figure 7:**
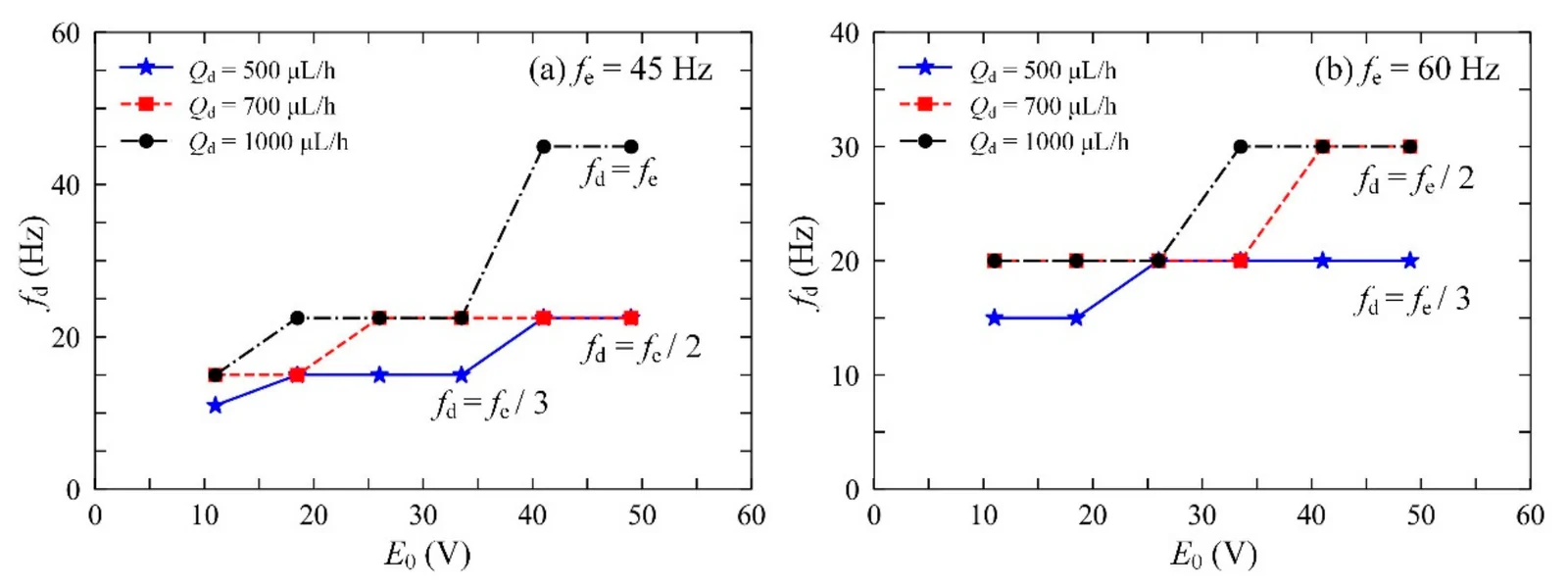
Effect of the excitation voltage on the droplet generation frequency (*Q*_c_ = 700 µL/h). (**a**) *f*_e_ = 45 Hz, (**b**) *f*_e_ = 60 Hz.

Figure 7 presents the dependence of droplet generation frequency on excitation voltage. The excitation voltage is varied from *E*_0_ = 11 V to *E*_0_ = 49 V. Two excitation frequencies are chosen, i.e., *f*_e_ = 45 Hz and *f*_e_ = 60 Hz. It can be observed that the droplet generation frequency exhibits a step increase with the increase in the excitation voltage for both *f*_e_ = 45 Hz and 60 Hz. For example, under excitation at *f*_e_ = 45 Hz, the droplet generation frequency for *Q*_d_ = 1000 µL/h rises from *f*_d_ = 15 Hz at *E*_0_ = 11 V to *f*_d_ = 22.5 Hz at *E*_0_ = 18.5 V and then remains unchanged until *E*_0_ = 33.5 V. Further, the droplet generation frequency increases to *f*_d_ = 45 Hz at *E*_0_ = 41 V and 45 V. Note that the subharmonic synchronization of *f*_d_ = *f*_e_/2 occurs at *E*_0_ = 18.5 ~ 33.5 V for *Q*_d_ = 1000 µL/h, *E*_0_ = 26 ~ 49 V for *Q*_d_ = 700 µL/h, and *E*_0_ = 41 ~ 49 V for *Q*_d_ = 500 µL/h. The minimal voltage for *f*_d_ = *f*_e_/2 reduces with the increasing flow rate of the dispersed phase. Under the excitation of *f*_e_ = 60 Hz, the droplet generation frequency for *Q*_d_ = 1000 µL/h is *f*_d_ = 20 Hz at *E*_0_ = 11 ~ 26 V to *f*_d_ = 30 Hz at *E*_0_ = 33.5 ~ 49 V. With the increase in the excitation voltage, the disturbance on the flow rate of the continuous phase is enhanced. The rising flow rate of the continuous phase would accelerate the neck thinning rate in squeezing mode, under the effect of the enlarged squeezing force [8]. As the excitation voltage increases beyond a critical value, one droplet is formed from every three excitation periods to every two excitation periods. As a result, the frequency synchronization varies from *f*_d_ = *f*_e_/3 to *f*_d_ = *f*_e_/2. In the numerical simulations of droplet formation with pulsatile flow of the continuous phase [52], the droplet generation frequency remains unchanged with the increase in the excitation amplitude. The step rising tendency is not observed in their work, which may be due to the small width ratio of the continuous phase channel to the dispersed phase channel.

**Figure 8:**
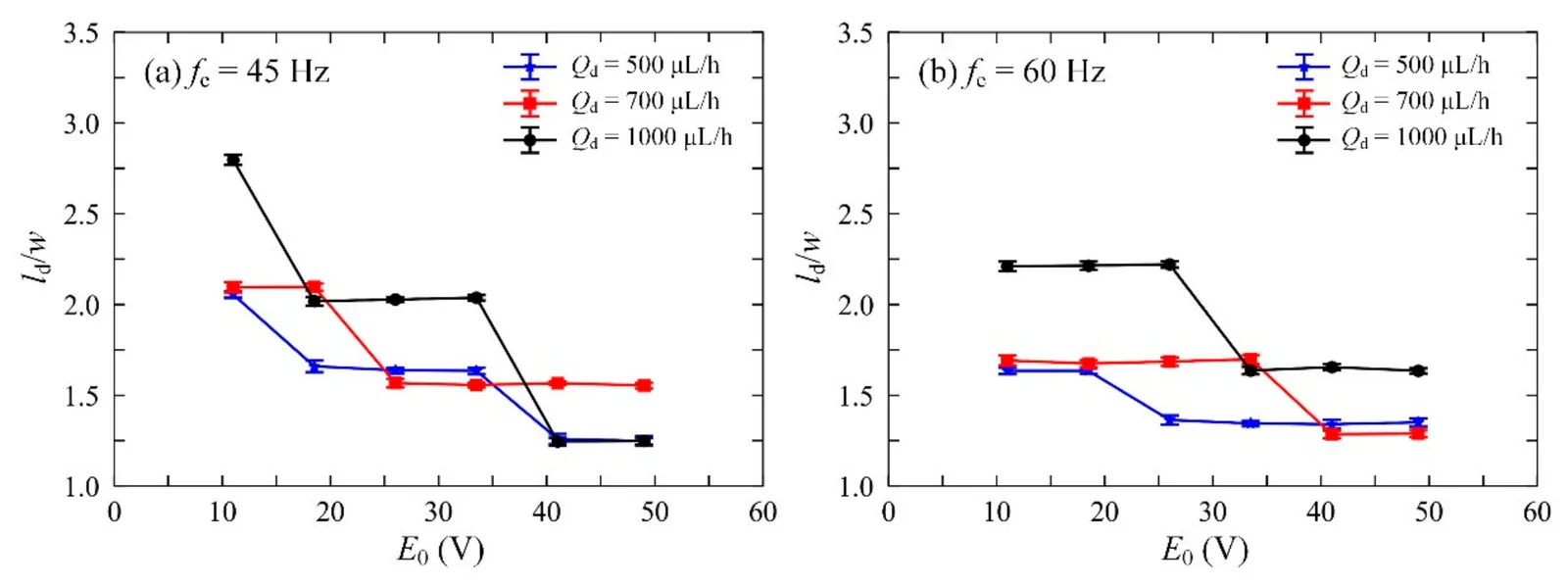
Effect of the excitation voltage on the droplet length (*Q*_c_ = 700 µL/h). (**a**) *f*_e_ = 45 Hz, (**b**) *f*_e_ = 60 Hz.

Figure 8 presents the average value and standard deviation of the dimensionless droplet length at various excitation voltages. The flow rate of the dispersed phase is varied from *Q*_d_ = 500 µL/h to *Q*_d_ = 1000 µL/h, and the droplet generation is excited at *f*_e_ = 45 Hz and *f*_e_ = 60 Hz, respectively. It can be seen that the droplet length exhibits a step reduction with the increase in the excitation voltage. Specifically, under excitation at *f*_e_ = 45 Hz, the droplet length for *Q*_d_ = 1000 µL/h reduces from *l*_d_*/w* = 2.80 at *E*_0_ = 11 V to *l*_d_*/w* ≈ 2.02 at *E*_0_ = 18.5 ~ 33.5 V, and further decreases to *l*_d_*/w* ≈ 1.25 at *E*_0_ = 41 ~ 49 V. The droplet length for *Q*_d_ = 700 µL/h reduces from *l*_d_*/w* ≈ 2.09 at *E*_0_ = 11 ~ 18.5 V to *l*_d_*/w* ≈ 1.56 at *E*_0_ = 26 ~ 49 V. The droplet length for *Q*_d_ = 500 µL/h reduces from *l*_d_*/w* = 2.05 at *E*_0_ = 11 V to *l*_d_*/w* ≈ 1.64 at *E*_0_ = 18.5 ~ 33.5 V, and further decreases to *l*_d_*/w* ≈ 1.26 at *E*_0_ = 41 ~ 49 V. Under excitation at *f*_e_ = 60 Hz, the droplet length for *Q*_d_ = 1000 µL/h reduces from *l*_d_*/w* ≈ 2.21 at *E*_0_ = 11 ~ 26 V to *l*_d_*/w* ≈ 1.64 at *E*_0_ = 33.5 ~ 49 V. With the increase in the excitation voltage, the step reduction in the droplet length can be ascribed to the step increase in the droplet generation frequency. In the numerical simulations of droplet formation with pulsatile flow of the continuous phase [52], excitation amplitude has a weak impact on droplet volume. In contrast, in the investigation of droplet formation with the dispersed phase excitation, droplet volume rises linearly with excitation voltage [54]. The difference between the present work and previous studies may be ascribed to the microchannel geometry and flow conditions.

### 3.3. Evolution and Mechanism of the Droplet Formation in the Synchronization Mode

The droplet length and generation frequency can be controlled by adjusting the excitation frequency and the excitation voltage of the piezoelectric actuators. Within the synchronization mode, the droplet generation frequency equals the excitation frequency and its subharmonics, e.g., *f*_d_ = *kf*_e_, where *k* = 1, 1/2, and 1/3. To explore the physical mechanism behind this, the evolution of the droplet formation for the regimes of *f*_d_ = *f*_e_ and *f*_d_ = *f*_e_/2 is examined in this section.

**Figure 9:**
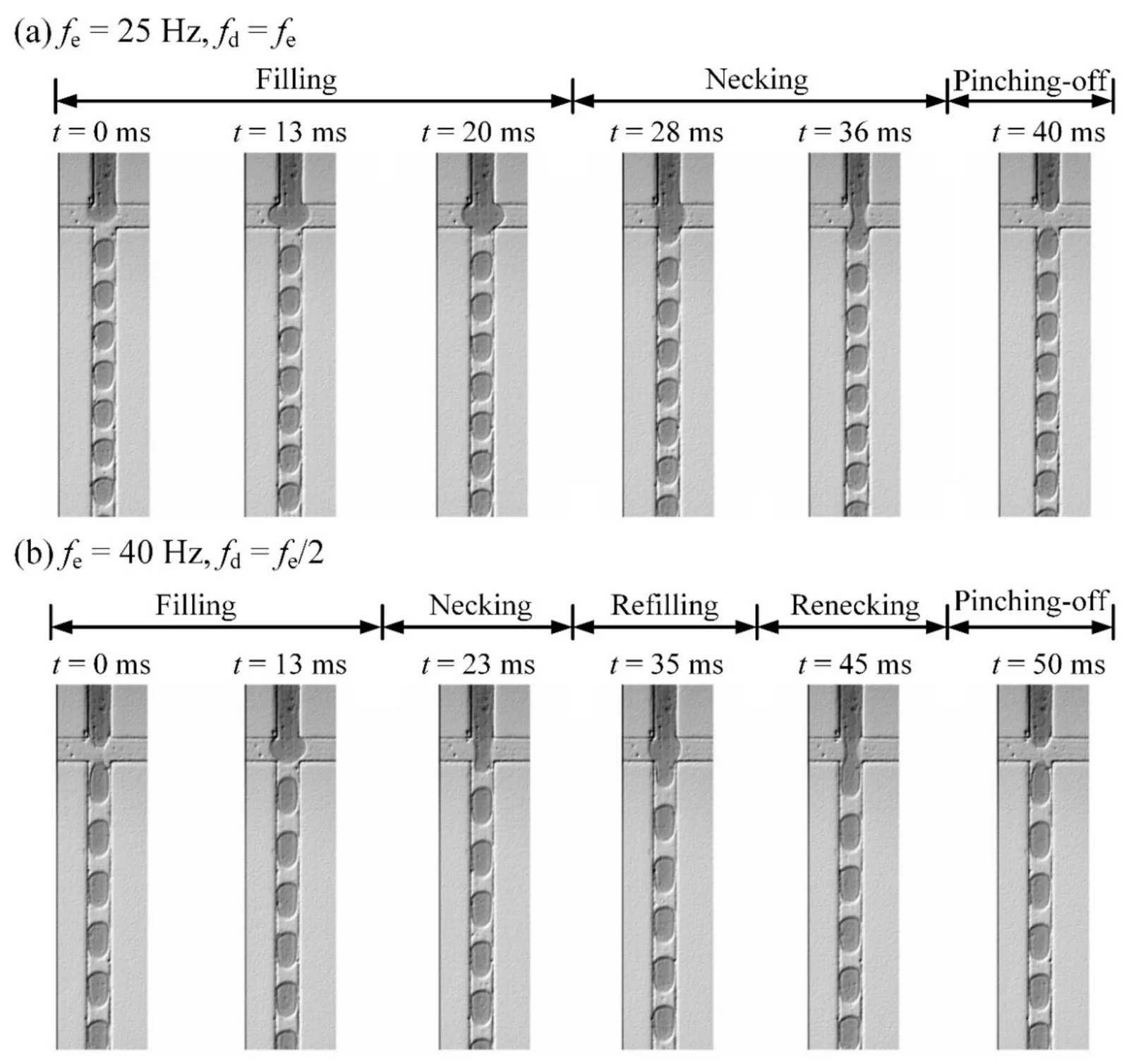
Evolution of droplet generation in the synchronization mode (*Q*_d_ = *Q*_c_ = 700 µL/h, *E*_0_ = 40 V). (**a**) *f*_e_ = 25 Hz, *f*_d_ = *f*_e_ and (**b**) *f*_e_ = 40 Hz, *f*_d_ = *f*_e_/2.

Figure 9 presents sequential photographs of the droplet formation process at *f*_e_ = 25 Hz and *f*_e_ = 40 Hz, corresponding to *f*_d_ = *f*_e_ and *f*_d_ = *f*_e_/2, respectively. The flow rates of the dispersed phase and the continuous phase are fixed at *Q*_d_ = *Q*_c_ = 700 µL/h, and the excitation voltage is chosen as *E*_0_ = 40 V. Under excitation of *f*_e_ = 25 Hz, droplet formation undergoes three stages, i.e., filling, necking, and pinching-off. Note that the head of the dispersed phase at the end of the filling stage fully blocks the continuous phase in the cross-junction. The incoming flow of the continuous phase would squeeze the droplet neck until the droplet is pinched off. This is similar to droplet formation in squeezing mode without excitation [33,55,56]. However, there is a slight difference from the unexcited counterparts in the filling stage, that is, the head of the dispersed phase expands into two upstream channels of the continuous phase at the filling stage. This is due to the reduced flow rate of the continuous phase in the descending stage of each excitation period. Under excitation at *f*_e_ = 40 Hz, the droplet generation frequency is half the excitation frequency. This indicates that one droplet is generated every two periods of excitation. The droplet generation undergoes five stages, i.e., filling, necking, refilling, re-necking, and pinching-off. Note that there is a gap between the head of the dispersed phase and the channel wall at the necking stage. This indicates that the head of the dispersed phase is not large enough to block the continuous phase during the first period of the excitation. Thus, the dispersed phase continues to fill the cross-junction. Further, the head of the dispersed phase fully blocks the continuous phase at the refilling stage. As a result, the accumulated pressure caused by the incoming flow of the continuous phase would squeeze the neck and finally generate a droplet.

According to the Hagen–Poiseuille law, the flow rate *Q* in a microchannel is linearly related to the pressure drop Δ*p*, i.e., *Q* = Δ*p*/*R*_h_, where *R*_h_ is the flow resistance [57]. Therefore, the pressure change Δ*p*_cj_ at the junction caused by the flow rate fluctuation could be expressed by

(10) $$\Delta p_{\mathrm{cj}}=Q_{e}(t)R_{\mathrm{hc}}=2\pi f_{e}Ad_{33}E_{0}R_{\mathrm{hc}}\cos(2\pi f_{e}t)$$

where *R*_hc_ is the flow resistance along the continuous phase channel.

The pressure change could lead to a variation in the flow rate of the dispersed phase at the junction. Therefore, the total flow rate of the dispersed phase at the junction can be calculated by

(11) $$Q_{d}(t)=Q_{d}+\frac{2\pi f_{e}Ad_{33}E_{0}R_{\mathrm{hc}}\cos(2\pi f_{e}t)}{R_{\mathrm{hd}}}$$

where *Q*_d_ is the flow rate at the inlet of the dispersed phase, and *R*_hd_ is the flow resistance along the dispersed phase channel.

If a droplet is generated at a frequency of *f*_d_, the droplet volume can be calculated by

(12) $$V_{d}=\int_{0}^{\frac{1}{f_{d}}}Q_{d}(t)dt=\int_{0}^{\frac{1}{f_{d}}}[Q_{d}+\frac{2\pi f_{e}Ad_{33}E_{0}R_{\mathrm{hc}}\cos(2\pi f_{e}t)}{R_{\mathrm{hd}}}]dt$$

In the synchronization mode, we have *f*_d_ = *kf*_e_, where *k* = 1, 1/2, and 1/3. The result of Equation (12) is given as

(13) $$V_{d}=\frac{Q_{d}}{f_{d}}=\frac{Q_{d}}{kf_{e}}$$

where the coefficient *k* depends on the excitation frequency and voltage. Equation (13) indicates that the droplet volume is inversely proportional to the excitation frequency in the synchronization mode. The change in *k* is due to the transition between three synchronization regimes, which may be responsible for the step variation of the droplet length with the increasing voltage.

As shown in Figure 9, the head of the dispersed phase grows in the filling stage. If the head volume could fully block the continuous phase, the droplet would eventually pinch off. Therefore, the head volume *V*_head_ should be larger than a critical value *V*_block_.

(14) $$\begin{array}{cc} V_{\mathrm{head}} & =\int_{0}^{\Delta t_{\mathrm{filling}}}Q_{d}(t)dt=\int_{0}^{\Delta t_{\mathrm{filling}}}[Q_{d}+\frac{2\pi f_{e}Ad_{33}E_{0}R_{\mathrm{hc}}\cos(2\pi f_{e}t)}{R_{\mathrm{hd}}}]dt\\ & =Q_{d}\Delta t_{\mathrm{filling}}+\gamma E_{0}\sin(2\pi f_{e}\Delta t_{\mathrm{filling}})\geq V_{\mathrm{block}} \end{array}$$

where Δ*t*_filling_ is the duration time of the filling stage, and the coefficient *γ* = *Ad*_33_*R*_hc_/*R*_hd_. The sinusoidal function in the expression of *V*_head_ suggests that the effect of the excitation voltage is related to the excitation period. As *E*_0_ decreases beyond a critical value, such that *V*_head_ < *V*_block_, more excitation periods are required to block the continuous phase. This is the reason why the droplet generation frequency exhibits step reductions with decreasing excitation voltage. As *f*_e_ increases, Δ*t*_filling_ reduces, and the first term *Q*_d_Δ*t*_filling_ in Equation (14) decreases. Once *V*_head_ < *V*_block_, more excitation periods are required to generate a droplet. This is responsible for the subharmonic phenomenon of the droplet generation frequency and the excitation frequency. As the excitation frequency further rises, the duration of each cycle is much shorter than the filling time of the dispersed phase, and the effect of the excitation frequency becomes more weakened. As a result, the synchronization mode is absent.

### 3.4. Regime Diagram of the Droplet Generation Frequency

As discussed in previous sections, droplet formation could be altered by controlling the excitation voltage and frequency of the stacked piezoelectric actuators. When the excitation frequency *f*_e_ is below a critical value, the droplet generation synchronizes with the piezoelectric excitations, producing monodispersed droplets. The droplet generation frequency equals the excitation frequency and its subharmonics, i.e., *f*_d_ = *f*_e_, *f*_d_ = *f*_e_/2, and *f*_d_ = *f*_e_/3. The boundaries between these regimes depend on the excitation frequency and voltage.

**Figure 10:**
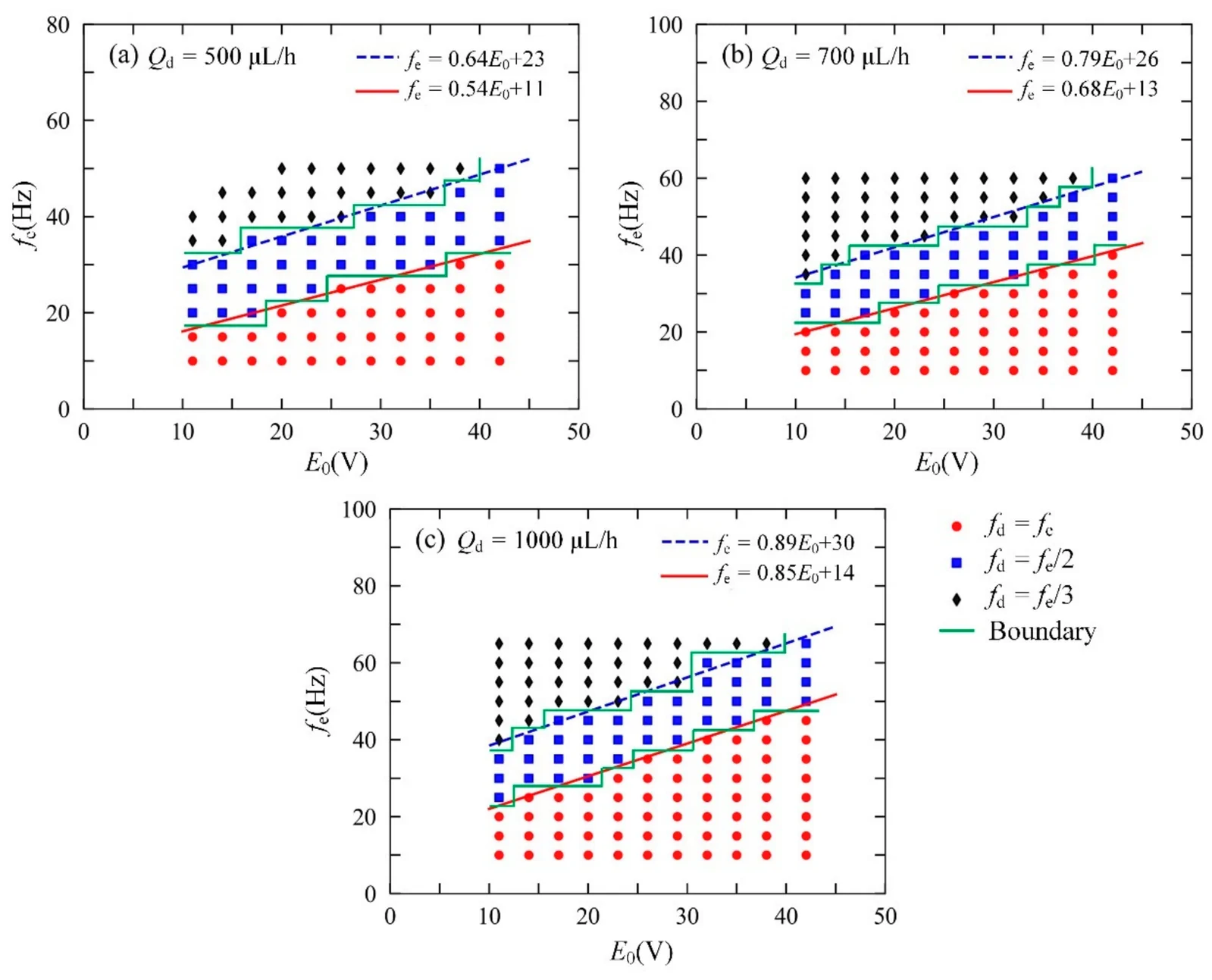
Regime diagram of the droplet generation frequency in synchronization mode (*Q*_c_ = 700 µL/h).

Figure 10 presents the regime diagram of the droplet formation frequency at different flow rates of the dispersed phase. The excitation frequency varies from *f*_e_ = 10 Hz to *f*_e_ = 65 Hz while the excitation voltage changes from *E*_0_ = 11 V to *E*_0_ = 42 V. It can be observed that the droplet formation frequency under the excitations has three regimes, i.e., *f*_d_ = *f*_e_, *f*_d_ = *f*_e_/2, and *f*_d_ = *f*_e_/3. Although the boundaries among these regimes exhibit a step increase with the rising excitation voltage, they could be approximately fitted with a linear scaling. At *Q*_d_ = 500 µL/h, the boundaries among the regimes of *f*_d_ = *f*_e_, *f*_d_ = *f*_e_/2 and *f*_d_ = *f*_e_/3 are described by *f*_e_ = 0.54*E*_0_ + 11 and *f*_e_ = 0.64*E*_0_ + 23, respectively. The boundaries among the three regimes at *Q*_d_ = 700 µL/h are given by *f*_e_ = 0.68*E*_0_ + 13 and *f*_e_ = 0.79*E*_0_ + 26 while those at *Q*_d_ = 1000 µL/h are expressed by *f*_e_ = 0.85*E*_0_ + 14 and *f*_e_ = 0.89*E*_0_ + 30. Therefore, the boundaries among each regime of the droplet generation frequency can be roughly given by the scaling of *f*_e_ = *αE*_0_ + *β*, where *α* and *β* depend on the flow rate of the dispersed phase. To achieve good control of droplet formation using piezoelectric actuators, it is suggested that the excitation voltage and frequency be chosen to ensure a droplet generation frequency within the regime of *f*_d_ = *f*_e_.

### 3.5. Scaling of the Generated Droplet Length

For a long droplet travelling in a square microchannel, the relationship between the droplet length *l*_d_ and the droplet volume *V*_d_ can be approximately expressed by

(15) $$V_{d}=\chi l_{d}w^{2}-\xi$$

where *χ* and *ξ* are coefficients, and *w* is the channel width.

By substituting Equation (15) into Equation (7), we have

(16) $$\frac{l_{d}}{w}=A\frac{Q_{d}}{w^{3}f_{d}}+B$$

where $A=\frac{1}{\chi }$ and $B=\frac{\xi }{\chi w^{3}}$. This suggests that the dimensionless droplet length is proportional to the flow rate of the dispersed phase and inversely proportional to the droplet generation frequency.

**Figure 11:**
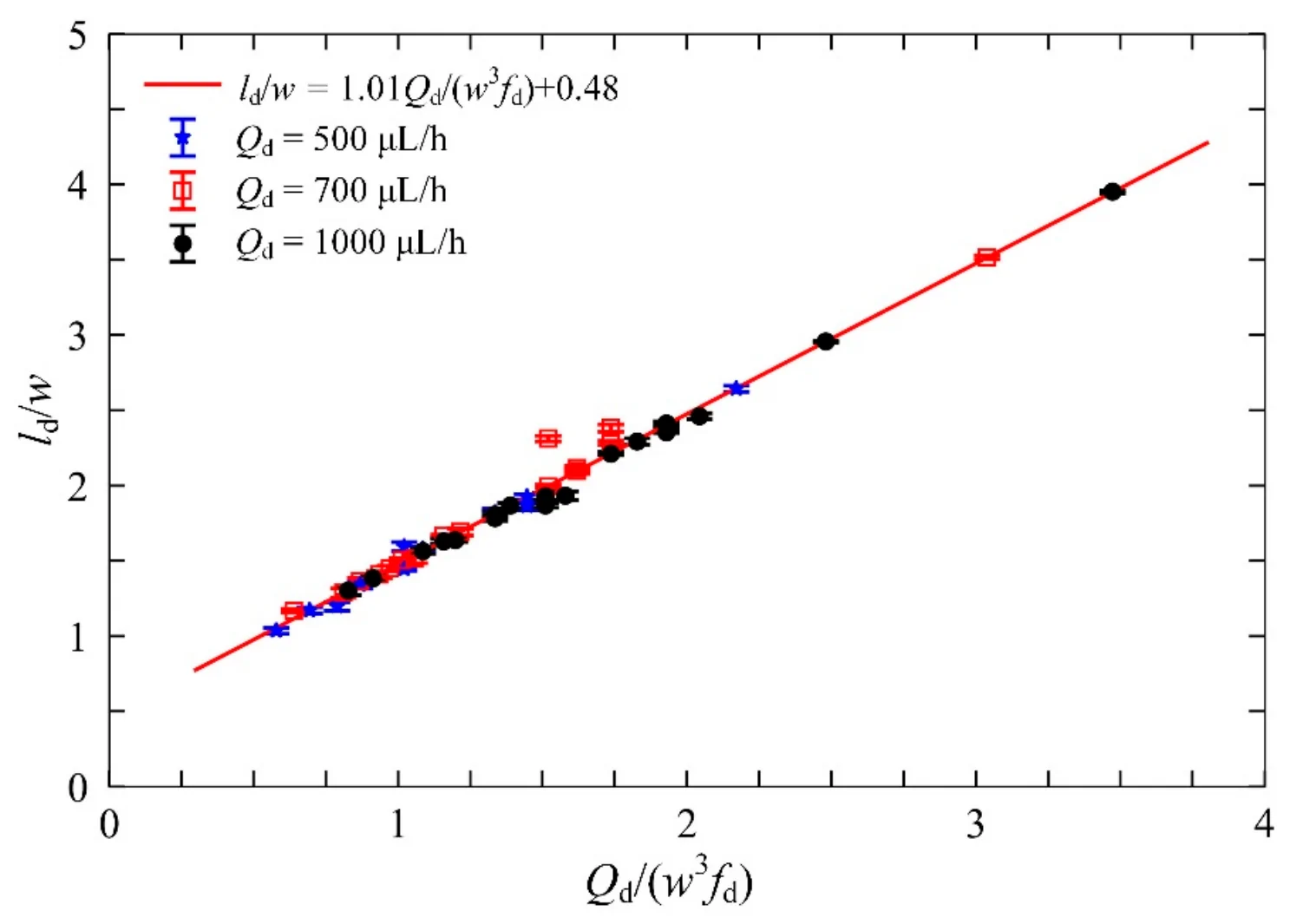
Scaling of the droplet length with the droplet generation frequency (*Q*_c_ = 700 µL/h).

Figure 11 presents a scaling of the dimensionless droplet length at various flow rates of the dispersed phase. The flow rate of the dispersed phase increases from 500 µL/h to 1000 µL/h, and the excitation frequency varies from *f*_e_ = 8 Hz to *f*_e_ = 200 Hz. It can be seen that all data collapse onto a linear scaling of $\frac{l_{d}}{w}=1.01\frac{Q_{d}}{w^{3}f_{d}}+0.48$. Note that the droplet generation frequency is linearly related to the excitation frequency in the synchronization mode, i.e., *f*_d_ = *kf*_e_, where *k* = 1, 1/2, and 1/3. It is suggested that the piezoelectric actuators be operated in the region of *f*_d_ = *f*_e_, where the droplet generation frequency range is larger than that in other regimes. Consequently, the generated droplet length can be adjusted based on the scaling of $\frac{l_{d}}{w}=1.01\frac{Q_{d}}{w^{3}f_{e}}+0.48$.

## 4. Conclusions

This work presents an experimental investigation on the active control of droplet formation in a cross-junction with continuous phase excitations. Two stacked piezoelectric actuators are placed adjacent to the continuous phase channels, producing flow fluctuations of the continuous phase. The dependence of the droplet formation on the excitation frequency and voltage of the piezoelectric actuators is examined in detail, and the evolution of droplet formation in synchronization mode is unveiled. The main conclusions can be drawn as follows.

(1) Droplet formation exhibits a dominant dependence on the excitation frequency of the piezoelectric actuators. The droplet generation frequency *f*_d_ is linearly related to the excitation frequency *f*_e_ in the synchronization mode, i.e., *f*_d_ = *kf*_e_, where *k* = 1, 1/2, and 1/3. Beyond a critical value, the effect of excitation frequency on droplet formation can be neglected. The droplet generation frequency can be increased up to ~2.6 times that without excitation.

(2) The droplet generation frequency exhibits a stepwise increase with the rising excitation voltage. Correspondingly, the droplet length decreases in a stepwise manner with the increase in the excitation voltage. The stepwise variations can be ascribed to the transitions between synchronization regimes.

(3) Droplet formation under excitation is mainly in the squeezing mode. The evolution of droplet formation for *f*_d_ = *f*_e_ includes three stages, i.e., filling, necking, and pinching-off. In contrast, droplet formation for *f*_d_ = *f*_e_/2 undergoes additional stages of refilling and re-necking. This is because the head of the dispersed phase at the junction during the first period of the excitation is not large enough to block the continuous phase.

(4) The regime diagram for the droplet generation frequency in synchronization mode is presented. The boundaries among three regimes of *f*_d_ = *f*_e_, *f*_d_ = *f*_e_/2, and *f*_d_ = *f*_e_/3 exhibit a step increase with the rising excitation voltage. In spite of this, the boundaries can be approximately fitted by the equation *f*_e_ = *αE*_0_ + *β*, where *α* and *β* depend on the flow rate of the dispersed phase.

(5) Droplet formation can be effectively controlled in the synchronization mode. The droplet length is proportional to the flow rate of the dispersed phase and inversely proportional to the droplet generation frequency, given by a scaling of $\frac{l_{d}}{w}=1.01\frac{Q_{d}}{w^{3}f_{d}}+0.48$.

In this work, the mass flow rate of the continuous phase, viscosity ratio, and interfacial tension are fixed. Future work could investigate the effect of the capillary number, viscosity ratio, and interfacial tension on the scaling law of droplet generation.

## Figures and Tables

**Table 1:** Fluid properties of the dispersed and continuous phases.

Fluids	Viscosity *μ* (Pa∙s)	Density *ρ* (kg/m^3^)	Interfacial Tension *σ* (N/m)
Liquid paraffin	16.07 × 10^−3^	0.84 × 10^3^	46.7 × 10^−3^
Distilled water	0.89 × 10^−3^	1.0 × 10^3^	

## Data Availability

The original contributions presented in this study are included in the article. Further inquiries can be directed to the corresponding author(s).

## References

[1] Lan F., Demaree B., Ahmed N., Abate A.R. (2017). Single-cell genome sequencing at ultra-high-throughput with microfluidic droplet barcoding. Nat. Biotechnol..

[2] Lareau C.A., Duarte F.M., Chew J.G., Kartha V.K., Burkett Z.D., Kohlway A.S., Pokholok D., Aryee M.J., Steemers F.J., Lebofsky R. (2019). Droplet-based combinatorial indexing for massive-scale single-cell chromatin accessibility. Nat. Biotechnol..

[3] Wang S., Liu Y., Li Y., Lv M., Gao K., He Y., Wei W., Zhu Y., Dong X., Xu X. (2022). High-throughput functional screening of Antigen-specific T cells based on droplet microfluidics at a single-cell level. Anal. Chem..

[4] Shen Y., Ruggeri F.S., Vigolo D., Kamada A., Qamar S., Levin A., Iserman C., Alberti S., George-Hyslop P.S., Knowles T.P.J. (2020). Biomolecular condensates undergo a generic shear-mediated liquid-to-solid transition. Nat. Nanotechnol..

[5] McCall P.M., Kim K., Shevchenko A., Ruer-Gruß M., Peychl J., Guck J., Shevchenko A., Hyman A.A., Brugués J. (2025). A label-free method for measuring the composition of multicomponent biomolecular condensates. Nat. Chem..

[6] Šneiderienė G., González Díaz A., Adhikari S.D., Wei J., Michaels T., Šneideris T., Linse S., Vendruscolo M., Garai K., Knowles T.P.J. (2025). Lipid-induced condensate formation from the Alzheimer’s Aβ peptide triggers amyloid aggregation. Proc. Natl. Acad. Sci. USA.

[7] Shang L., Cheng Y., Zhao Y. (2017). Emerging droplet microfluidics. Chem. Rev..

[8] Zhu P., Wang L. (2017). Passive and active droplet generation with microfluidics: A review. Lab Chip.

[9] Ding Y., Howes P.D., deMello A.J. (2020). Recent advances in droplet microfluidics. Anal. Chem..

[10] Chen Z., Kheiri S., Young E.W.K., Kumacheva E. (2022). Trends in droplet microfluidics: From droplet generation to biomedical applications. Langmuir.

[11] Yang H., Knowles T.P.J. (2022). Hydrodynamics of droplet sorting in asymmetric acute junctions. Micromachines.

[12] Yang H., Xu Y., Knowles T. (2023). Droplet dynamics in asymmetric microfluidic junctions. Eur. J. Mech. B-Fluids.

[13] Nan L., Zhang H., Weitz D.A., Shum H.C. (2024). Development and future of droplet microfluidics. Lab Chip.

[14] Jena S.K., Bahga S.S., Kondaraju S. (2021). Prediction of droplet sizes in a T-junction microchannel: Effect of dispersed phase inertial forces. Phys. Fluids.

[15] Kumari P., Atta A. (2022). Insights into the dynamics of non-Newtonian droplet formation in a T-junction microchannel. Phys. Fluids.

[16] Marcali M., Chen X., Aucoin M.G., Ren C.L. (2022). Droplet formation of biological non-Newtonian fluid in T-junction generators. I. Experimental investigation. Phys. Rev. E.

[17] Pang Y., Yang Q., Wang X., Liu Z. (2022). Dripping and jetting generation mode in T-junction microchannels with contractive structures. Phys. Fluids.

[18] Zhang J., Zhang X., Zhao W., Liu H., Jiang Y. (2022). Effect of surfactants on droplet generation in a microfluidic T-junction: A lattice Boltzmann study. Phys. Fluids.

[19] Jena S.K., Srivastava T., Bahga S.S., Kondaraju S. (2023). Effect of channel width on droplet generation inside T-junction microchannel. Phys. Fluids.

[20] Wang Z., Xiang X., Zhu H., Dong Y., Zhu C., Ma Y., Sun B., Patlazhan S.A., Fu T. (2023). Generation of droplets of shear-thinning non-Newtonian fluids in T-junction parallelized microchannels. Chem. Eng. J..

[21] Li S., Xiang X., Wang Z., Zhu C., Ma Y., Fu T. (2024). Uniformity and stability of droplet formation at T-junctions in symmetrical microchannels. Chem. Eng. J..

[22] Liu Z., Su P., Pang Y., Liu W., Wang Z., Wang X. (2023). Dynamic mechanism of double emulsion droplets flowing through a microfluidic T-junction. Phys. Fluids.

[23] Chen Y., Wu L., Zhang L. (2015). Dynamic behaviors of double emulsion formation in a flow-focusing device. Int. J. Heat Mass Transf..

[24] Azarmanesh M., Farhadi M., Azizian P. (2016). Double emulsion formation through hierarchical flow-focusing microchannel. Phys. Fluids.

[25] Chen X., Ren C.L. (2017). Experimental study on droplet generation in flow focusing devices considering a stratified flow with viscosity contrast. Chem. Eng. Sci..

[26] Lashkaripour A., Rodriguez C., Ortiz L., Densmore D. (2019). Performance tuning of microfluidic flow-focusing droplet generators. Lab Chip.

[27] Nazari M., Varedi-Koulaei S.M., Nazari M. (2022). Flow characteristics prediction in a flow-focusing microchannel for a desired droplet size using an inverse model: Experimental and numerical study. Microfluid. Nanofluidics.

[28] Liu Y., Zhu C., Fu T., Gao X., Ma Y. (2024). Breakup dynamics of water-in-water droplet generation in a flow-focusing microchannel. Chem. Eng. Sci..

[29] Yang H., Knowles T.P.J. (2026). Flow behavior of a droplet in viscous fluids through asymmetric bifurcating microchannels. Phys. Fluids.

[30] Wu J., Yan Q., Cui Y., Xuan S., Gong X. (2017). Geometry-confined bifurcation at low flow rate in flow-focusing droplet generator. Microfluid. Nanofluidics.

[31] Chong Z.Z., Tan S.H., Gañán-Calvo A.M., Tor S.B., Loh N.H., Nguyen N.-T. (2016). Active droplet generation in microfluidics. Lab Chip.

[32] Fu T., Wu Y., Ma Y., Li H.Z. (2012). Droplet formation and breakup dynamics in microfluidic flow-focusing devices: From dripping to jetting. Chem. Eng. Sci..

[33] Chen X., Glawdel T., Cui N., Ren C.L. (2014). Model of droplet generation in flow focusing generators operating in the squeezing regime. Microfluid. Nanofluidics.

[34] Nooranidoost M., Izbassarov D., Muradoglu M. (2016). Droplet formation in a flow focusing configuration: Effects of viscoelasticity. Phys. Fluids.

[35] Du W., Fu T., Duan Y., Zhu C., Ma Y., Li H.Z. (2018). Breakup dynamics for droplet formation in shear-thinning fluids in a flow-focusing device. Chem. Eng. Sci..

[36] Roumpea E., Kovalchuk N.M., Chinaud M., Nowak E., Simmons M.J.H., Angeli P. (2019). Experimental studies on droplet formation in a flow-focusing microchannel in the presence of surfactants. Chem. Eng. Sci..

[37] Lashkaripour A., Rodriguez C., Mehdipour N., Mardian R., McIntyre D., Ortiz L., Campbell J., Densmore D. (2021). Machine learning enables design automation of microfluidic flow-focusing droplet generation. Nat. Commun..

[38] Sheng L., Zheng C., Chang Y., Deng J., Luo G. (2025). Bubble generation in a 3-D flow-focusing microchannel: From squeezing to jetting. Chem. Eng. Sci..

[39] Garstecki P., Fuerstman M.J., Stone H.A., Whitesides G.M. (2006). Formation of droplets and bubbles in a microfluidic T-junction—Scaling and mechanism of break-up. Lab Chip.

[40] Liu H., Zhang Y. (2011). Droplet formation in microfluidic cross-junctions. Phys. Fluids.

[41] Xu J.H., Li S.W., Tan J., Luo G.S. (2008). Correlations of droplet formation in T-junction microfluidic devices: From squeezing to dripping. Microfluid. Nanofluidics.

[42] Shi N., Mohibullah M., Easley C.J. (2021). Active flow control and dynamic analysis in droplet microfluidics. Annu. Rev. Anal. Chem..

[43] Teo A.J.T., Yan M., Dong J., Xi H.-D., Fu Y., Tan S.H., Nguyen N.-T. (2020). Controllable droplet generation at a microfluidic T-junction using AC electric field. Microfluid. Nanofluidics.

[44] Liu Z., Cai F., Pang Y., Ren Y., Zheng N., Chen R., Zhao S. (2022). Enhanced droplet formation in a T-junction microchannel using electric field: A lattice Boltzmann study. Phys. Fluids.

[45] Wang D., Chagot L., Wang J., Angeli P. (2025). Effect of electric field on droplet formation in a co-flow microchannel. Phys. Fluids.

[46] Yang Q., Wang Z., Gao Q., Zhao Y., Jiang C. (2024). Study of droplet formation in parallel flow focusing microchannel under electrostatic field control. Colloids Surf. A Physicochem. Eng. Asp..

[47] Liang D., Ma R., Fu T., Zhu C., Wang K., Ma Y., Luo G. (2019). Dynamics and formation of alternating droplets under magnetic field at a T-junction. Chem. Eng. Sci..

[48] Kholardi M.D., Farhadi M. (2024). Understanding droplet formation in T-shaped channels with magnetic field influence: A computational investigation. Phys. Fluids.

[49] Zhu P., Tang X., Wang L. (2016). Droplet generation in co-flow microfluidic channels with vibration. Microfluid. Nanofluidics.

[50] Tang S.Y., Ayan B., Nama N., Bian Y., Lata J.P., Guo X., Huang T.J. (2016). On-chip production of size-controllable liquid metal microdroplets using acoustic waves. Small.

[51] Zhao S., Liu Z., Zheng N., Zhang C., Zheng K., Shi S., Pang Y. (2025). Study on the size and spatial configuration of liquid metal droplets in conductive hydrogels induced by surface acoustic waves. Lab Chip.

[52] Duan L., Yuan W., Liu D., Chen F., Wei J. (2025). Active control of complex non-Newtonian polymer droplet formation in flow-focused microchannels by pulsatile flow. Chem. Eng. Sci..

[53] Lu K., Huang B., Chen Z., Wang W., Yang H. (2025). Droplet generation in a flow-focusing microchannel with continuous phase perturbations. Eur. J. Mech. B-Fluids.

[54] Bransky A., Korin N., Khoury M., Levenberg S. (2009). A microfluidic droplet generator based on a piezoelectric actuator. Lab Chip.

[55] Yu W., Liu X., Zhao Y., Chen Y. (2019). Droplet generation hydrodynamics in the microfluidic cross-junction with different junction angles. Chem. Eng. Sci..

[56] van Loo S., Stoukatch S., Kraft M., Gilet T. (2016). Droplet formation by squeezing in a microfluidic cross-junction. Microfluid. Nanofluidics.

[57] Beebe D.J., Mensing G.A., Walker G.M. (2002). Physics and applications of microfluidics in biology. Annu. Rev. Biomed. Eng..

